# Current practices in the use of sildenafil for pulmonary arterial hypertension in Brazilian hospitals

**DOI:** 10.1186/1756-0500-2-30

**Published:** 2009-03-02

**Authors:** Ana HA de Souza, Lucio M Cabral, Gisele Huf

**Affiliations:** 1 Pharmacy service, National Institute of Cardiology, Rio de Janeiro, Brazil; 2 Pharmaceutical Industrial Tecnology Lab, Federal University of Rio de Janeiro, Rio de Janeiro, Brazil; 3 National Institute of Quality Control in Health, Oswaldo Cruz Foundation, Rio de Janeiro, Brazil

## Abstract

**Background:**

Sildenafil is a cyclic guanosine monophosphate-specific phosphodiesterase-5 inhibitor used for treating pulmonary hypertension. Although the use of sildenafil in patients under 18 years old is off-label, this inhibitor has been widely prescribed for children treatment at hospitals in Brazil. In this work we evaluated the current practices in using sildenafil in the three main reference hospitals of Rio de Janeiro to design a clinical trial. Then we analyzed the content of sildenafil in powder paper preparations used in these institutions.

**Methods and Results:**

We assessed the data about the use of sildenafil in three reference hospitals including Instituto Nacional de Cardiologia – INC, Instituto Estadual de Cardiologia Aloysio de Castro – IECAC and Hospital Pro-Cardíaco – HPC. The pharmacy records were analyzed from April 1st, 2008 to April 30th, 2008 and interviews with the pharmacists were also performed. Our results showed that INC used the greatest amount of sildenafil in: treatment of pulmonary arterial hypertension (PAH), management of transient PAH during surgery, preparation for cardiac transplantation and haemodynamic studies during cardiac catheterization. Meanwhile IECAC and HPC used sildenafil only for treating PAH in few patients during the period evaluated. In INC and IECAC, sildenafil was available in tablets, and powder papers prepared by two private pharmacies and one public hospital pharmacy. In contrast all patients of HPC received sildenafil in tablets with no external manipulation. Our quantification analysis results using reverse-phase high performance liquid chromatography method showed that powder papers prepared by the private pharmacies from the sildenafil tablets presented only 58.5 to 89.3% of the declared concentration in contrast to samples from the public hospital pharmacy (104.4 to 105.3%).

**Conclusion:**

Few patients received the prescribed sildenafil dose at the reference hospitals evaluated in Rio de Janeiro, which may importantly compromise this inhibitor effect in the current treatment. This study reinforced the need of checking the practices of preparing and administering sildenafil continually.

## Background

Sildenafil is a cyclic guanosine monophosphate-specific phosphodiesterase-5 inhibitor used for the management of pulmonary hypertension. According to the Brazilian guidelines the dosage established for pulmonary arterial hypertension (PAH) is 20 mg three times a day at the beginning of the treatment and 0.3 mg/kg/day to 3 mg/kg/day divided in three doses for pediatric patients [1]. Despite the existence of other treatment options such as oral endothelin antagonists and prostacyclin analogues (inhaled, subcutaneous or intravenous), they are not available in Brazil due to the expensive cost [2].

Sildenafil might be an effective pulmonary vasodilator as suggested by several case reports [3-6] and some trials in children [7,8]. In most of these studies, the dose of sildenafil was 2 mg/kg every 6 hours. Sildenafil has also been used during cardiac catheterization to evaluate the haemodynamic deterioration and the vascular bed response to pharmacologic stimuli. During this procedure any degree of reversibility is searched, which provides important prognostic information [9]. The haemodynamic study is usually performed to identify children who are responsive to vasodilators as a part of preoperative assessment to congenital heart disease correction. Vasoreactive patients have a better long-term prognosis and are more likely to respond favorably to the pharmacologic treatment than non-responsive patients and children with transient PAH during surgery. The Cochrane systematic review about the use of sildenafil in adults suffering from PAH showed that only four clinical trials with 77 participants met the inclusion criteria [10].

The Brazilian agency for drug regulation, the National Agency of Sanitary Surveillance (ANVISA), released the use of sildenafil for erectile disfunction in 1998 and PAH treatment in 2006. In both cases the license was only approved for patients above 18 years old in tablets with 25, 50 and 100 mg of sildenafil. Since it is the most available option in Brazil, it has been widely prescribed to patients of all ages at Brazilian hospitals. The situation leads to some problems as some inpatients (*i.e. *nasogastric or nasoenteric tube-fed patients, those with altered mental status, and small children) require treatment with oral liquid dosage forms that are not frequently available.

In the absence of oral liquid dosage forms and to overcome the swallowing problems, usually three practices are performed at the Brazilian hospitals that include: cutting sildenafil tablets into smaller segments (halves or quarters), openning sildenafil capsules, and using sildenafil injectable solutions for oral administration. It is also common the preparation of extemporaneous powders by redistributing sildenafil from commercially available crushed tablets or opened capsules into powder papers (sachets) after using diluents (*i.e. *lactose).

Currently, the only advantage for children treatment using sildenafil powder papers instead of tablets is the easier swallowing process. However there are several limitations involved in this use including:

• Errors during the preparation of the powder papers (*i.e. *content homogeneity) especially when using crushed tablets. A worse situation is noticed when, in the lack of the powder papers, tablets are directly crushed by non-appropriate staff (i.e. nurses) for using on patients.

• Dose accuracy since sildenafil solubility in water is 1 mg/3.5 ml and usually the powder paper is diluted in 5 ml of water. Since the minimum amount of water to dilute one powder paper containing 5 mg of sildenafil is 17.5 ml, probably part of the drug is not in solution, and the children are not getting the entire drug amount that is necessary.

• Stability of the formulation that does not allow the drug to be stored to posterior administration. On that matter, there is a lack of stability studies of sildenafil powder paper in water.

• Amount of water to dilute each powder paper to children with water restriction (*i.e. *17.5 ml of water to dilute 5 mg of sildenafil).

• Powder papers diluents (*i.e. *lactose) that may cause diarrhea, dehydration and metabolic acidosis in children with lactose intolerance [11].

For all these reasons, studies about sildenafil should consider the practices of preparing and administering this drug. Therefore, in this work we evaluated the current practices of using sildenafil in three main reference cardiologic hospitals of Rio de Janeiro to design a clinical trial. Then we analyzed the content of sildenafil present in the powder paper used in these hospitals.

## Methods

Rio de Janeiro has approximately 6 million habitants, and two public reference cardiologic hospitals (Instituto Nacional de Cardiologia-INC and Instituto Estadual de Cardiologia Aloysio de Castro – IECAC), which are responsible for attending 70% of the population, and a private reference cardiologic hospital (Hospital Pro-Cardíaco – HPC) that works only with private health insurance patients. Our survey analyzed the pharmacy records of these three reference hospitals searching for everyone who had used sildenafil during the period of April 1st, 2008 to April 30th, 2008. In this study, the main pharmacist of each institution was also interviewed for additional information about the use of sildenafil in clinical conditions and drug preparation for children use. The protocol of this study was approved by the Ethics Committee of Instituto Nacional de Cardiologia.

Since the preparation of the powder papers used by the two public hospitals (INC and IECAC) was performed by two private pharmacies and one public hospital pharmacy, we collected five powder papers from each pharmacy to be randomly selected and tested for quality control. In this analysis, we used the reverse-phase high performance liquid chromatography (HPLC) method developed by Equifarma for quantification of sildenafil in the powder papers. Briefly, the powder packs were diluted with the mobile phase where for each powder paper, two samples were produced and each sample was analyzed twice. The HPLC column was a LiChrospher C18 (5 μm) 25 × 0.46 cm using the ammonium acetate 0.2 M pH 7.0:acetonitrile (1:1) as mobile phase. The injection volumes were 20 μl and the flow rate was 1 ml/min. The column was monitored at 240 nm using a UV detector wavelength.

## Results

### Instituto Nacional de Cardiologia (INC) – overview

Instituto Nacional de Cardiologia (INC) is a public institution for cardiac surgery located at Rio de Janeiro. In 2000, due to its services quality level, INC became a reference center for guidelines in cardiology area. Currently INC is responsible for most of the pediatric cardiac surgeries of the state of Rio de Janeiro.

During the period of our evaluation, there were 5402 emergency attendances, 368 inpatients and 253 ambulatorial attendances. Sildenafil has been used at INC since December 2004 prescribed for treating pulmonary arterial hypertension (PAH), restoring transient PAH during cardiac surgery, cardiovascular stabilization to heart transplantation and in haemodynamic study during cardiac catheterization. Importantly, during 2005 and 2006 more than 70 children had at least one prescription of sildenafil.

The usual dosage of sildenafil for treating PAH in pediatric patients is 0.3 to 0.4 mg/kg/dose every 8 hours [12] and 0.2 mg/kg in haemodynamic study with cardiac catheterization. Since the hospital contains sildenafil in 25 mg-tablets and considering the low weight of the children eligible for the treatment and procedures, these tablets are routinely sent to a private pharmacy to prepare 5 mg-sildenafil powder papers. Then these powder papers are sent to the nurses that add 5 ml of water and calculate the required amount to be administered.

### Instituto Estadual de Cardiologia Aloysio de Castro (IECAC) – overview

Instituto Estadual de Cardiologia Aloysio de Castro (IECAC) is a reference state hospital that offers diagnosis services and treatment for cardiovascular diseases. IECAC is of great importance for the public health of Rio de Janeiro state due to its high-qualified staff that treats different and complex cardiovascular disorders. During the period of our evaluation, there were 3583 outpatients attendances, 20 adult cardiac surgeries, 8 pediatric surgeries, 134 cardiac and vascular catheterizations and 834 imaging studies.

Similar to INC, sildenafil is also available at IECAC prescribed to adults and children with PAH. In this hospital, sildenafil tablets are also sent to a private pharmacy for preparing 1 mg-sildenafil powder papers.

### Hospital Pro-Cardíaco (HPC) – overview

Hospital Pro-Cardíaco (HPC) is a reference in cardiologic assistance in Rio de Janeiro since the inauguration in 1959. During all these years, it has improved its performance by contracting different cardiac disorders specialists, developing researches and using a technology comparable to the best health centers in the world.

During the period of our evaluation there were 203 emergency attendances with 136 inpatients admitted and no outpatients attendances. Sildenafil is also prescribed to adults and children in this hospital with no external manipulation, different from the other two hospitals. In fact sildenafil tablets are usually sent to the nurses in their original packs.

### Comparison of the use of sildenafil in children and adults in INC, IECAC and HPC

(see Additional file 1) shows that during the period of evaluation, INC widely used sildenafil for treatment of PAH, managing transient PAH during surgery, preparing for cardiac transplantation and as a vasodilator drug for haemodynamic study during cardiac catheterization. According to the pharmacist, once both pharmaceutical forms (tablets and powder papers) are available at this hospital, the clinical staff is free to decide the prescription (*i.e. *powder papers to adults with swallowing difficulties and tablets to older children).

During the period of evaluation, few patients used sildenafil at IECAC and HPC (see Additional file 1). Despite sildenafil is also available at IECAC in both pharmaceutical forms, the powder papers are only dispensed by the pharmacy to pediatric patients. According to the pharmacist, children who do not require manipulation of the commercial form may receive tablets. All patients at HPC received sildenafil tablets with no external manipulation.

### Sildenafil powder papers quantification analyses

The results of the quantification of sildenafil in the powder papers from two private pharmacies (A and B) and a public hospital pharmacy (C) used in the reference hospitals evaluated are shown in (see Additional file 2). The analysis showed that samples from A and B (58.5–89.3%) were below the minimum amount required of sildenafil (90–110%) whereas the public hospital pharmacy met the proper quality criteria (see Additional file 2).

## Discussion

The use of sildenafil is not well established in pulmonary arterial hypertension (PAH) due to the lack of evidence and poor methodologic quality studies. This situation is partially explained by the low prevalence of the pathology in the population and turn analyses about current practices in using this inhibitor of great interest. In Brazil, the hospitals data are not organized and collecting information is not an easy task. Thus surveys are difficult to carry on as also involve the analyses and combination of particularly fragmented records with the professionals point of view. In a developing world environment, this process may be even more problematic as vastly under-resource services and fully paper-based records that use different notation patterns are also frequent. For example, despite of obtaining all records on the use of sildenafil in the three reference hospitals evaluated, it is not possible to guarantee that the patients used all pills as this was not recorded on the pharmacy notes.

The three hospitals evaluated in this work are the main cardiologic care institutions at the state of Rio de Janeiro. They perform most of the treatment of cardiologic diseases in Rio de Janeiro. As most of the patients in use of sildenafil for PAH are assisted by these three institutions in this state, we suggested that the procedures observed in this study may reflect the reality for all patients.

Although we did not look into patients notes for the actual dose received by each patient, the protocol for using sildenafil in at least one hospital (INC) recommended a smaller dose (0.3 to 0.4 mg/kg/dose every 8 hours) than that described by some case reports (2 mg/kg/dose every 6 hours) [3,4]. It is of current knowledge that modifying a commercially available drug is always problematic. This modification may lead to increased toxicity, undesirable side effects, decreased efficacy, poor patient compliance due to medication taste and potential hazards to health care workers. Furthermore, this practice does not guarantee the right dose administration as errors may occur in preparing and administering the doses whereas the powder papers preparation are time-consuming, and difficult to make.

Considering that the acceptable percentage of a specific dose in a formulation is 90 to 110%, our data showed that none of the powder papers prepared by the pharmacies that were tested in this study would be considered appropriate in a quality control analysis, except by the public hospital pharmacy. This study suggested that most of patients who need treatment or diagnosis at these institutions are possibly not receiving the proper sildenafil dose, and it is difficult to predict the consequences of this practice for these patients. In addition it is possible to speculate that this practice is not only taking place in Rio de Janeiro, but possibly in more developed countries.

## Conclusion

Currently the use of sildenafil in children is affected by the crushing-tablets practice that leads to dosage errors. According to our results very few patients received the prescribed dose at some of these reference hospitals in the period analyzed. A trial was designed to compare the sildenafil powder papers regularly used in these hospitals with a sildenafil suspension developed following the good practices requirements of a compounding formulation, which provided good evidences for further studies in this under researched area.

## Competing interests

The authors declare that they have no competing interests.

## Authors' contributions

AHAdS – worked with other authors to draft the protocol, collected and analyzed the data and wrote the paper. LMC – worked with other authors to draft the protocol, helped on writing the paper. GH – worked with other authors to draft the protocol, analyzed the data and wrote the paper.

All authors read and approved the final manuscript.

## Supplementary Material

Additional file 1 **The use of sildenafil in three main reference hospitals of Rio de Janeiro – Instituto Nacional de Cardiologia (INC), Instituto Estadual de Cardiologia Aloysio de Castro (IECAC), and Hospital Pro-Cardíaco (HPC) – during April, 2008**. Data provided represent the amount of sildenafil (in mg) used in three hospitals in Rio de Janeiro during April, 2008. Click here for file

Additional file 2 **Quantification analysis results (%) of the sildenafil powder papers from two private pharmacies (A and B) and a public hospital pharmacy (C) that are suppliers of the reference hospitals**. The data provided represent the results of quantification analysis: the percentage of sildenafil present in the powder papers compounded by two private pharmacies (A and B) and a public hospital pharmacy (C) that are suppliers of the reference hospitals. Click here for file
