# Supplementary material for: Current practices in the use of sildenafil for pulmonary arterial hypertension in Brazilian hospitals
Source: BMC Res Notes. 2009 Mar 2;2:30. doi: 10.1186/1756-0500-2-30 (PMC2666750; doi:10.1186/1756-0500-2-30)
Supplement: Additional file 1 — The use of sildenafil in three main reference hospitals of Rio de Janeiro – Instituto Nacional de Cardiologia (INC), Instituto Estadual de Cardiologia Aloysio de Castro (IECAC), and Hospital Pro-Cardíaco (HPC) – during April, 2008. Data provided represent the amount of sildenafil (in mg) used in three hospitals in Rio de Janeiro during April, 2008. [file 1756-0500-2-30-S1.doc]

Table 1: The use of sildenafil in three main reference hospitals of Rio de Janeiro - Instituto Nacional de Cardiologia (INC), Instituto Estadual de Cardiologia Aloysio de Castro (IEAC), and Hospital Pro-Cardíaco (HPC) - during April, 2008.

| **Hospital** | **Clinical Conditions for**  **using Sildenafil** | Number of Patients using Sildenafil | | | | **Total** | | |
| --- | --- | --- | --- | --- | --- | --- | --- | --- |
|  |  | **Tablet** | | **Powder paper** | |  | **Patients**  (n) | **Sildenafil**  (mg) |
|  |  | Children | Adults | Children | Adults |  |  |  |
| INC | Pulmonary Arterial Hypertension (PAH),  Transient PAH during surgery,  Preparation for cardiac transplantation, Haemodynamic study - cardiac catheterization. | 5  (525mg*) | 11  (6675mg) | 10  (525mg) | 3  (125mg) |  | 29 | 7850 |
| IEAC | Pulmonary Arterial Hypertension (PAH) | 1  (225mg) | 2  (225mg) | 2  (54mg) | 0 |  | 5 | 504 |
| HPC | Pulmonary Arterial Hypertension (PAH) | 2  (575mg) | 0 | 0 | 0 |  | 2 | 575 |

*amount of sildenafil
