# Supplementary material for: Current practices in the use of sildenafil for pulmonary arterial hypertension in Brazilian hospitals
Source: BMC Res Notes. 2009 Mar 2;2:30. doi: 10.1186/1756-0500-2-30 (PMC2666750; doi:10.1186/1756-0500-2-30)
Supplement: Additional file 2 — Quantification analysis results (%) of the sildenafil powder papers from two private pharmacies (A and B) and a public hospital pharmacy (C) that are suppliers of the reference hospitals. The data provided represent the results of quantification analysis: the percentage of sildenafil present in the powder papers compounded by two private pharmacies (A and B) and a public hospital pharmacy (C) that are suppliers of the reference hospitals. [file 1756-0500-2-30-S2.doc]

Table 2: Quantification analysis results (%) of the sildenafil powder papers from two private pharmacies (A and B) and a public hospital pharmacy (C) that are suppliers of the reference hospitals.

| **Pharmacy** | **Powder Paper**  **Number** | **Sildenafil** | |
| --- | --- | --- | --- |
|  |  | (mg)^a^ | (%)^b^ |
| A | 01 | 1 | 73.7 |
|  | 02 | 1 | 85.2 |
|  | 03 | 1 | 89.3 |
| B | 04 | 5 | 58.5 |
|  | 05 | 5 | 67.4 |
| C | 06 | 5 | 105.3 |
|  | 07 | 5 | 104.4 |

a, b = declared dose and amount detected, respectively.
